# Portable Motion-Analysis Device for Upper-Limb Research, Assessment, and Rehabilitation in Non-Laboratory Settings

**DOI:** 10.1109/JTEHM.2019.2953257

**Published:** 2019-11-13

**Authors:** Won Joon Sohn, Rifat Sipahi, Terence D. Sanger, Dagmar Sternad

**Affiliations:** 1 Electrical & Computer Engineering and Physics DepartmentNortheastern University 27817 Boston MA 02115 USA; 2 Mechanical and Industrial Engineering DepartmentNortheastern University 27817 Boston MA 02115 USA; 3 Biomedical Engineering, Neurology, and Biokinesiology DepartmentUniversity of Southern California 5116 Los Angeles CA 90007 USA

**Keywords:** Quantitative motor assessment, kinematic data acquisition device, upper-limb movement disorder, cerebral palsy, stroke

## Abstract

This study presents the design and feasibility testing of an interactive portable motion-analysis device for the assessment of upper-limb motor functions in clinical and home settings. The device engages subjects to perform tasks that imitate activities of daily living, e.g. drinking from a cup and moving other complex objects. Sitting at a magnetic table subjects hold a 3D printed cup with an adjustable magnet and move this cup on the table to targets that can be drawn on the table surface. A ball rolling inside the cup can enhance the task challenge by introducing additional dynamics. A single video camera with a portable computer tracks real-time kinematics of the cup and the rolling ball using a custom-developed, color-based computer-vision algorithm. Preliminary verification with marker-based 3D-motion capture demonstrated that the device produces accurate kinematic measurements. Based on the real-time 2D cup coordinates, audio-visual feedback about performance can be delivered to increase motivation. The feasibility of using this device in clinical diagnostics is demonstrated on 2 neurotypical children and also 3 children with upper-extremity impairments in the hospital, where conventional motion-analysis systems are difficult to use. The device meets key needs for clinical practice: 1) a portable solution for quantitative motor assessment for upper-limb movement disorders at non-laboratory clinical settings, 2) a low-cost rehabilitation device that can increase the volume of in-home physical therapy, and 3) the device affords testing and training a variety of motor tasks inspired by daily challenges to enhance self-confidence to participate in day-to-day activities.

## I. Introduction

An integral part of clinical care for individuals with motor disorders is to assess motor function to guide and evaluate medical treatment, surgical intervention or physical therapy. One of the challenges for assessing motor function is to define sensitive and quantitative measures that can be readily obtained in clinical practice. The objective of this study was to develop a device that affords quantitative assessment of motor impairments in non-laboratory settings. The specific focus is on individuals with upper-limb movement disorders. One central goal was to ground the task in scientific research to relate clinical measures to research and capitalize on insights from fundamental research.

This paper first lays out the need for such a device particularly for children with motor disorders and post-stroke rehabilitation. We then motivate the specific motor task that was originally conceived for basic research on motor control. We then detail the design of the prototype with all hardware and software components so that it can be replicated. One design goal was to make the device low-cost, so that it can be used in many clinical environments including at home for therapeutic exercises. We conclude with first results from pilot experiments acquired both in a traditional laboratory setting and in an Epilepsy Monitoring Unit. These first data were obtained from children with dystonia. However, the device is not limited to this population and is currently further modified for the assessment of stroke patients.

### A. Clinical Assessments of Motor Disorders

A motor disorder manifests as an impaired ability to execute a movement with the intended spatial and temporal pattern. This includes abnormal posturing, presence of unintended excessive movement, and normal movements occurring at unintended or inappropriate times [1]. Patients with upper-limb impairments require special assistance to perform common motor tasks associated with self-care, such as feeding and dressing. Challenges in their movement control result in frustration, which leads to less engagement and practice, and thereby fewer opportunities to attenuate their motor disabilities and improve their movement control.

Motor disorder are observed also among children. Cerebral Palsy (CP) is a common cause of movement disorders among children, affecting 3 to 4 individuals per 1000 births in the US. The dyskinetic form of CP occurs in 15% of all cases [2]. Due to inflexible postures, caused by muscle spasms and contractures together with involuntary jerky movements, children with dyskinetic CP are often prevented from participation in many daily activities. This also prevents them from acquiring age-appropriate motor skills during critical periods of skill development [3], [4]. This is particularly aggravated when the condition affects the upper limbs.

For clinical motor assessments, the current standard tools are clinical scales. For cerebral palsy, typical tests are the gross motor function classification system (GMFCS) [5], the manual ability classification system (MACS) [6], the House Scale [7], the Melbourne Assessment [8], the Assisting Hand Assessment [9], the Hypertonia Assessment Tool (HAT) [10], the Barry-Albright Dystonia (BAD) scale [11], and the Shriners Hospital for Children Upper Extremity Evaluation [12]. These outcome measures were devised to satisfy the typical criteria for effective outcome measures, including reliability, validity, specificity, and responsiveness [13]. Although useful, these rating scales rely on subjective assessment and questionnaires that are vulnerable to inter-rater and test-retest reliability, nonlinearity, multi-dimensionality, and ceiling or floor effects [14]. These shortcomings need to be overcome by more quantitative outcome measures to provide a better evaluation of the individual’s motor functions and abilities, and potentially utilize such measures to objectvely assess and titrate interventions.

### B. Quantitative Assessment of Motor Function

Motion tracking technologies have provided quantitative means of recording movements through a variety of sensing technology that tracks and stores movement. Camera-based motion capture, such as Vicon (Vicon Motion Systems, Oxford, UK) and Optitrak (Northern Digital Inc, Ontario, CA) requires external markers or sensors placed on key anatomical landmarks to reconstruct the skeletal model of human body parts. These state-of-the-art technologies track motion to very high precision with high sampling rates and they have been used for pre- and post-treatment assessment of upper- or lower-extremity pathologies. However, such data acquisition is limited to traditional laboratory settings because the multi-camera systems are expensive and not portable.

On the other hand, there are low-cost inertial measurement units (IMUs) that directly measure acceleration, rotational change and magnetic orientation. While these sensors have the advantage that they are self-contained and wearable, drawbacks are degraded accuracy due to drift, calibration errors and noise inherent to inertial sensors and the need to frequently recharge batteries for real-time data streaming [15]. Moreover, attaching sensors to body parts can be inconvenient or even impossible for certain clinical populations, and many children will not tolerate them.

In view of the above arguments, there is a strong need for less invasive devices that can provide quantitative measurements in tasks related to upper-extremeity motor function. Preferably, such a device should allow for portability and be low-cost to reach large populations.

### C. Low-Cost Rehabilitation at Home

Rehabilitation follows standard practice and frequently requires one-on-one interaction with a therapist for extended periods of time. For these reasons, robotic devices have emerged to deliver higher-dosage and higher-intensity training for patients with movement disorders such as cerebral palsy and stroke [16]–[19]. However, while effective, robotic therapy is expensive and to date can only be used in clinical settings. To increase the volume in therapy, lower-cost devices that can be used at home are urgently needed.

Performance improvements with predominant home training are indeed possible. This was demonstrated by pediatric constraint-induced movement therapy (CIMT) for children with hemiparetic CP [20], [21]. Further, it was shown that even children with severe dystonia can improve their performance if they use an interface or device that enables and facilitates their severely handicapped movements [22].

A portable low-cost device for home use that is able to provide reliable quantitative measurements would help address the above shortcomings. Measurements could also be streamed to careproviders on a secure cloud protocol, for diagnosis of interventions, analysis of therapeutic outcomes, and further follow up.

### D. Theoretically-Grounded Rehabilitation

Motor tasks for home therapy should be engaging to avoid boredom and attrition and should also have functional relevance. With this goal in mind, we developed a motor task that was motivated by the daily self-feeding activity of leading a cup of coffee or a spoon filled with soup to the mouth. The core challenge of actions of this kind is that moving such an object with sloshing liquid presents complex interaction forces: any force applied to the cup also applies a force to the liquid that then acts back on the hand. When such internal dynamics is present, interaction forces become quite complex, and the human performing the task needs to predict and preempt the internal dynamics of the moving liquid. Clearly, better understanding task is like guiding a cup of coffee to one’s mouth or a spoonful of soup has high functional relevance. While many such functional tasks have been developed for rehabiltation (e.g., the box-and-block and the pegboard task), the quantitative assessment should allow for more than descriptive outcome measures such as error or success rate. Monitoring the ‘process’ continuously should provide more detailed insight into coordinative challenges. This is indeed possible in the task of guiding a cup of coffee as we explain next.

**FIGURE 1.**
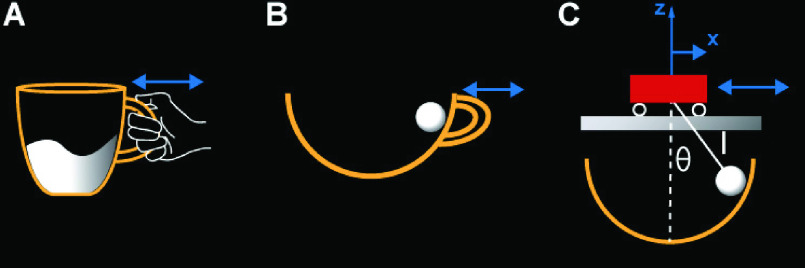
Model for the task of carrying a cup of coffee. A: The real object. B: The simplified physical model. C: The equivalent cart-and pendulum model implemented in the virtual task.

In previous research, we abstracted a relevant, yet simplified model task, inspired by guiding a cup of coffee [23]–[26]. To reduce the complexity and afford theoretical analyses, the “cup of coffee” was simplified to a rigid object with a rolling ball inside. The rolling ball represents the moving liquid; this is also similar to the children’s game of transporting an egg in a spoon [27]. Fig.1A-C shows the transition from the real object to the simplified physical model. Importantly, the original task (Fig.1A) was reduced to a two-dimensional model, where the subject interacts with the object via a robotic manipulandum. The virtual model consists of a cart with a suspended pendulum, a well-known benchmark problem in control theory.

Several studies have revealed that subjects learn through practice to make the complex object predictable by exploiting the resonance frequencies of the object or by stabilizing the cup trajectory [26], [28]. Several quantitative measures were developed that sensitively evaluated how subjects learn to handle the virtual cup-and-ball model. One continuous metric was a safety or energy margin that could sensitively quantify how safely subjects transported the virtual cup [23]. One study showed that older individuals moved the cup with smaller safety margins than younger people, but older individuals could also improve with practice [23].

**FIGURE 2.**
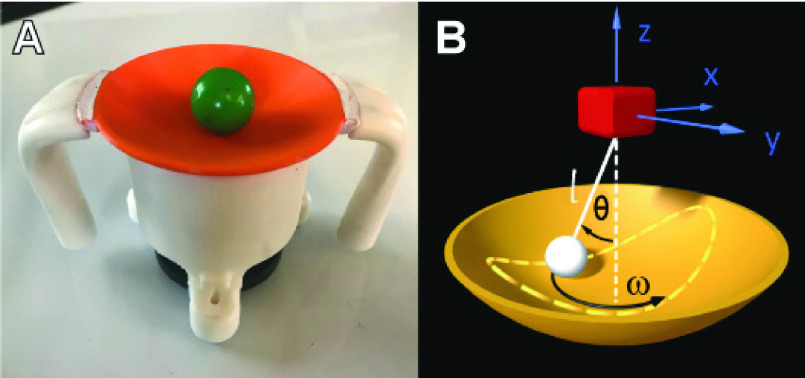
A: The 3D printed real object used on the MAGIC Table. B: The spherical pendulum as a model for the real cup and ball.

The virtual cup-ball model offers a foundation upon which one can expand to numerous investigations with therapeutic aims. While our past work was focused on understanding how healthy individuals manipulated the virtual cup, the underlying concepts can be extended to rehabilitation [29]. To this end, we developed a 3D physical version of this cup-and-ball system (Fig.2). In this custom-made 3D cup, a ball rolls inside the cup as shown in Fig.2A, mimicking the motion of a spherical pendulum (Fig.2B). The subject then moves the cup on a horizontal surface based on certain task objectives, e.g. moving to a target. The equations of motion for this cup-and-ball system are provided in the Appendix. They can be used to simulate trajectories of both the ball and the cup given any hypothetical time-varying input forces. They are also the basis for computing safety margins and other more theoretically-guided metrics [23].

### E. The Magic Table in Overview

**FIGURE 3.**
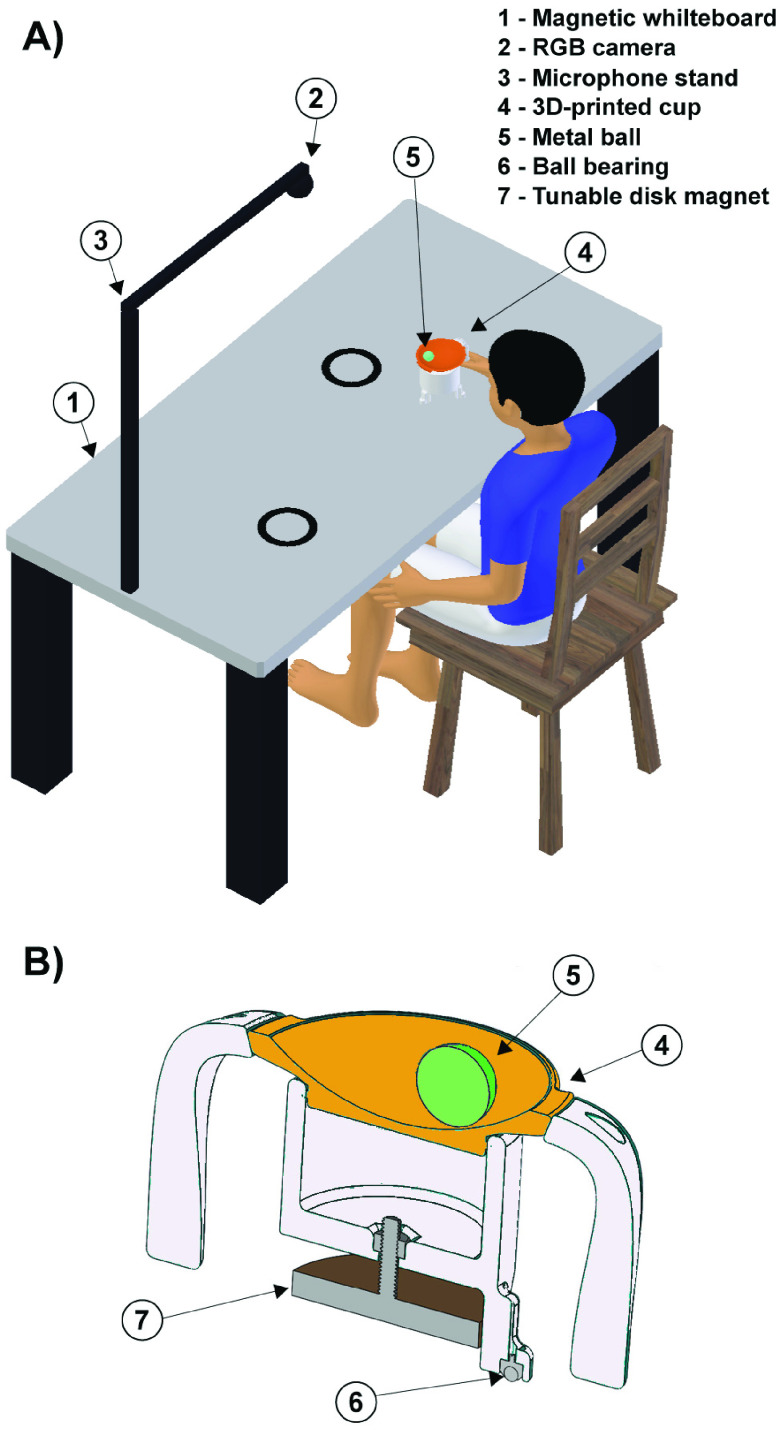
A: The MAGIC Table: Two circular targets are drawn on a magnetized whiteboard mounted on a table base to illustrate a task with point-to-point movement. A person is holding a magnetic cup while sitting on a chair in an upright position. A colored ball rolls in an orange-colored shallow dish. An RGB web camera is mounted on the stand that is fixed above the center of the board and adjusted to have a full table as its field-of-view. A mobile computer (now shown) is connected to the camera through USB 2.0 for the processing of the computer-vision-based motion-analysis. B: Design of the 3D-printed cup. A cross-sectional view of the cup visualizes the magnet and the metal ball that rolls on a removable shallow dish affixed atop the cup base.

The proposed device, the MAGnetic Interactive and Creative Table, or MAGIC Table, enables individuals with a wide spectrum of mild to severe upper-limb motor disorders to perform interactive movement tasks on a horizontal whiteboard (Fig.3). Subjects grasp a custom-made 3D-printed cup and slide it over a whiteboard which has a metallic plate underneath the surface (a typical magnetic dry-erase board). A magnet at the bottom of the cup provides assistance to the subjects and ensures to keep the cup in contact with the table surface. This magnet is connected to the cup by a bolt and its distance to the metallic surface determines the strength of the magnetic attraction. This distance can be adjusted to determine the appropriate balance between magnetic attraction and resistence to the horizontal movements. This design parameter is customizable to the need of the individual subjects. For example, persons with hypertonic movement disorder may benefit from higher magnetic attraction to keep the movement in the horizontal plane.

To mimick the challenge of transporting of a cup of coffee, a ball can be placed inside the cup that sensitively responds to the cup’s acceleration. To avoid losing the ball, subtle adjustments of the cup movements are required. To create different challenges, the cup can have different degrees of curvatures and different rim heights that increase the risk of losing the ball. The therapist, parent or the patient can draw targets on the whiteboard with a regular color marker.

The camera system comprises an RGB web camera connected to a mobile computer. The camrea is mounted above the table to track real-time kinematics of the hand-held cup and ball on the table surface. Using a color- and shape-based computer-vision algorithm, the motion of the cup and ball are tracked without attaching any markers to the body. The camera can also tracks targets together with the cup movements. This enables the creative design of a variety of drawing, tracking and targeting games. Motivational feedback is also available: the online measured 2D cup coordinates can be used to provide real-time, audio-visual feedback about performance quality, i.e., spatial and temporal errrors and safety margins, although not further elaborated in this paper [23]. Feedback or reward can be visually displayed on a screen in front of the user and/or can be played auditorily. A portable computer records the data on cloud networks that enable tele-rehabilitation.

Our device leverages new technology to address three key needs: 1) It is a portable solution for quantitative motor assessment for upper-limb movements in non-laboratory clinical settings. 2) It allows to create a suite of motor tasks that are inspired by daily motor challenges, such as self-feeding; hence improvements are likely to transfer to day-to-day activities. 3) When used as a home training device, it can increase the volume of in-home physical therapy. 4) It is grounded in theoretical analysis and affords an easy bridge between laboratory research and clinical field data.

### F. Summary of Technical Novelties

The novel features of the MAGIC Table are as follows:

• The specific motor task was motivated by our research on interactive movements. In previous work we created a virtual task based on a physical model that afforded several theoretically-guided metrics for evaluating motor control principles. This previous work provides a theoretical basis for the transition to clinical applications [29].
• The laboratory task was rendered into a real-world setting and the interactive task captures the essence of activities for daily living (ADL). Compared to learning in virtual environments, transfer of skill to the real everyday activities is less problematic [30].
• The MAGIC Table engages individuals in daily motor challenges such as self-feeding, which may develop self-confidence to participate in other activities. The whiteboard surface also allows an individualized creative design of tasks and games with color markers.
• The combination of the metallic table surface with the magnetized 3D-printed cup enables a variety of purposeful movement tasks, while also setting a gentle restraint on involuntary movements, characteristic of many neurological impairments. For many disorders this is preferable to hard physical constraints, as for example presented by exoskeletons.
• A markerless tracking system obviates the need to attach markers or other sensors to patients and opens up a new avenue for expedient data acquisition during patients’ routine visit to clinics.
• The device is portable and affords the flexible design of a variety of intuitive tasks, while also recording precise kinematics of movements.
• Highly accurate sensing affords quantitative assessment for diagnosis, analysis, and comparative studies.
• Simple procedures afford use in restricted clinical and home settings. Parallel use in laboratory research and clinical settings affords bridging therapeutic applications with research.
• Since the device poses no hard constraints and the motor challenges can be configured by the user, it is expected that the user will experience less frustration and will be motivated for a longer time, an important requisite to improve motor control.
• The MAGIC Table is light-weight to make it portable and suitable for home-based rehabilitation. Cost to produce the table is approximately $200, promising that the table can reach large populations. If the patient can exercise from home, this reduces therapist time and costs to the national health services.

## II. Details of Design, Methods and Procedures

This section details the hardware and the custom-developed software of the MAGIC Table.

### A. Hardware Construction

Fig.3A shows an overview of the MAGIC Table design. A magnetic whiteboard is affixed on top of a table base, set to a height facilitating arm movements across the table for the individual. Targets can be drawn on the table surface. A variable focal-length RGB web camera (ELP 2.8–12 mm Varifocal lens HD 1080P Webcam, PN: ELP-USBFHD01M-BFV) is fixed to a mounting device above the center of the table surface. Different versions of the MAGIC Table used a microphone stand with a tripod base or a scissor arm. Its height can be adjusted to have the full table in its field-of-view. For the prototype used for the pilot data collection it was set to 105 cm above the table surface. A mobile computer was connected to the camera through USB 2.0 for processing of the computer-vision-based motion analysis.

Fig.3B illustrates the cross-sectional view of the 3D-printed cup. The cup is affixed atop of a base with three legs. The legs are installed with low-friction 8-mm-thick carbon steel nylon ball bearing (TOMUM, CY-8H-NL). The tuning of the magnetic attraction between the cup and table is achieved by adjusting the distance between magnet and surface (Fig.3B, McMaster PN: 7132T25, Diameter: 2.57”). A green steel ball (BC Precision, PN: BCRASST, Diameter: 1.00, mass: 67 g), shown as a disc in cross-section, rolls in the orange shallow dish. The colors of the cup and ball were selected to facilitate color-based tracking. The cup has two detachable ergonomic handles, similar to a sippy cup, that can be used for both unimanual and bimanual control. For the pilot experiment, the height of the magnet was set to 5 mm above the board, which afforded attraction of the cup to the surface while minimizing resistance to horizontal movements.

**FIGURE 4.**
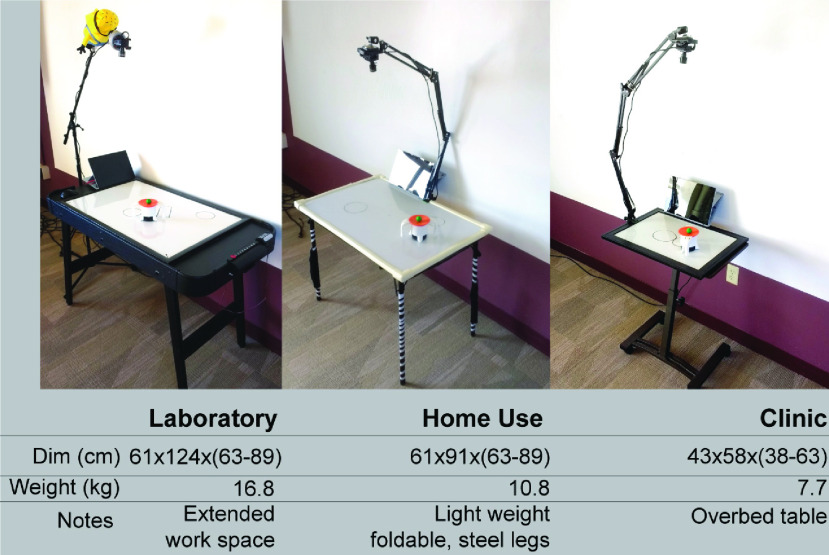
Three prototype implementations of a table depending on size restrictions.

Different versions of the table were designed. The first table was developed for the initial laboratory testing. The height of the table was 70 cm (27.5”) and the dimensions of the board were 60.9 $\times$ 91.4 cm (24 $\times$ 3”). The table base was a commercially available game table. A second table was constructed to comply with airline size specifications as it was transported to a hospital at a distant location. The four legs were foldable to facilitate portability. The weight of the prototype including table, camera stand, camera, cup was 10.8 kg. A third table was constructed for use at the bedside in the hospital. Specifications are provided in Fig.4. All table versions can be constructed with ready-made parts from home vendors.

### B. Surface Board Registration

The RGB webcam is connected through USB 2.0 to a mobile computer (Microsoft Surface Pro 4, Microsoft Corp., Redmond, US). For consistent registration of the surface board, the camera’s field-of-view is manually adjusted by a 5-point matching process to ensure a consistent field-of-view across trials: 5 points on the table are matched to corresponding points on a virtual template of the board (the 4 points on the vertices of the rectangular field of view and 1 point at the crossing of the diagonals in the center should coincide with the whiteboard dimension). This registration can be repeated if the camera location is disturbed during use. After the board registration, a snapshot of the camera view is taken with the circular targets drawn on the board.

**FIGURE 5.**
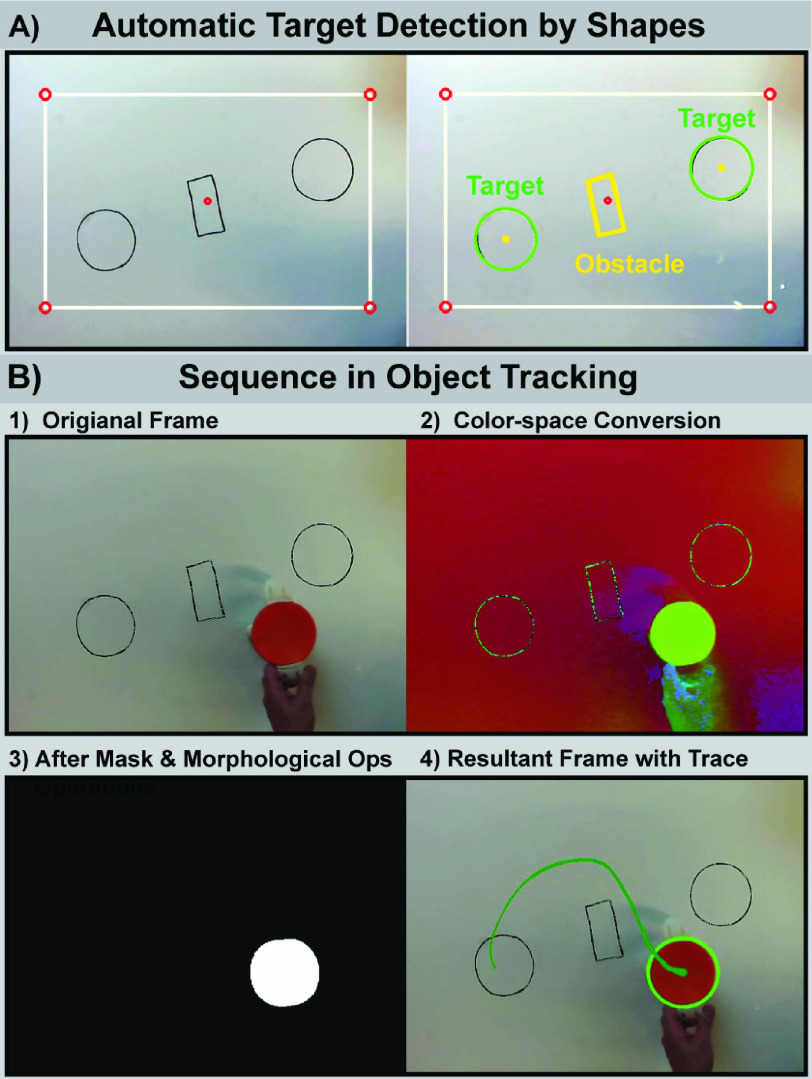
A: Automatic target detection by shapes. Left: original frame. Right: targets are designated as circles and obstacles as polygons. Obstacles are not shown here for demonstration and were not used in the study. B: Target registration image processing with computer vision. 1) Raw image from an RGB camera. 2) Color space conversion from RGB to HSV. 3) After morphological operations and masking. 4) Resultant frame with the trajectory of the center of the cup (green).

To assure accurate coordinates of the targets, a transparent plastic sheet of the size of the board with pre-drawn targets covered the surface to assure the same task parameters across participants. Target detection and registration were automatized with the computer-vision algorithm HoughCircles in OpenCV library (Fig.5A).

### C. Computer-Vision-Based Object Tracking

The position of the cup is tracked with a series of computer-vision algorithms that are applied frame by frame as received from the camera. The frame rate for a resolution of 640$\times$480 is 80 fps with Surface Pro 4 (Microsoft Surface Pro 4 tablet PC, i5-6300U, 8GB RAM) and 150 fps with Desktop PC (Intel Core i7-7700 CPU @3.60GHz 16G RAM). Spatial precision for the current implementation is 0.75m/640 pixels = 1.4 mm.

Fig.5B shows the key steps for the computer-vision-based detection of the cup position. After a frame is received from the camera, the color space of the image is converted from RGB to HSV for easier color representation. Next, a HSV mask is applied to extract spatial regions of the cup by color; subsequently, morphological operations of erosion and dilation are applied. Erosion is applied with a 2D convolution kernel of 5$\times$5 pixels to remove low levels of white noise; dilation is used to magnify the representation of the object of interest, which is reduced by the erosion. Next, all contours in the current image frame are searched and sorted to find the best fit ellipse for the circular cup contours to locate the center of the cup. A starter software code package for real-time object tracking in python is provided in the online repository (https://github.com/wonjsohn/MAGIC_Table_basic).

### D. Model of the Task and Dependent Measures

As mentioned previously, the task was inspired by the daily task of self-feeding, but also by the basic motor challenge of interacting with complex objects. In previous work, we developed an experimental paradigm using a virtual environment with a robotic interface. In several studies, we showed that controlling a complex object with rich dynamics, such as a cup with a rolling ball inside, poses challenges that go beyond reaching and pointing [22], [24], [26], [31].

The current MAGIC Table version of the task is a 3D version of the previous simplified 2D model (Fig.2B). Extending from the cart-and-pendulum model, where the cart moves on a horizontal line and the pendulum has one degree of freedom, the equations of motion were extended to those of a spherical pendulum, suspended to a cart moving on a horizontal surface. The equations with spherical coordinates are provided in the Appendix.

To allow model-based analyses of the measured data it is important that the position and velocities measured from the cup and the ball in the real MAGIC Table map directly onto the variables of the model system. The mass of the cup and the ball map directly onto parameters in the equations, and pendulum length is represented by the curvature of the cup.

### E. Manipulating Task Difficulty

To vary the level of challenge for the motor task, several parameters of the object can be easily created by 3D-printing of different cups: 1) curvature of the dish in which the metal ball rolls, 2) size and mass of the cup, 3) size and mass of the ball. 4) strength of magnetic attraction of the cup. Going beyond the modeled task, a safety rim placed on the perimeter of the dish can be added to decrease likelihood of escape of the ball. Evidently, the task can also be performed without the rolling ball inside.

In addition, a wide variety of task scenarios can be drawn on the whiteboard. Targets or obstacles can be drawn at will, because the camera can recognize polygons. With this ability, the caregiveers can draw their own targets and create games for children. Further, as described before, the camera can record online the displacement of the cup and ball, together with the targets drawn on the table. Hence, relevant measures can be online calculated in the computer and certain signals can then be fed back to the subject. These features afford engaging games for children such that boredom and the resulting attrition can be attenuated.

### F. Environment and Data Processing

A portable tablet PC (Surface Pro 4 tablet PC, i5-6300U, 8GB RAM) was selected to conduct the pilot experiment. Python (3.6), PyQt5 (5.12), PyOpenGL (3.1.0), imutils (0.4.6), opencv-python (3.1.0) were the main libraries required to import. A PyQt-based graphical user interface (GUI) was developed for the user-friendly operation in data collection. More information about the GUI can be found in the online repository. Performance improvement was achieved by utilizing dual-threading, one for the camera frame retrievals and the other for the main loop to prevent delays by blocking operations. Kinematic data were recorded in python pandas dataframe library (pandas.DataFrame 0.23.4) and videos were recorded as well.

### G. Preliminary Verification of Accuracy of Data

**FIGURE 6.**
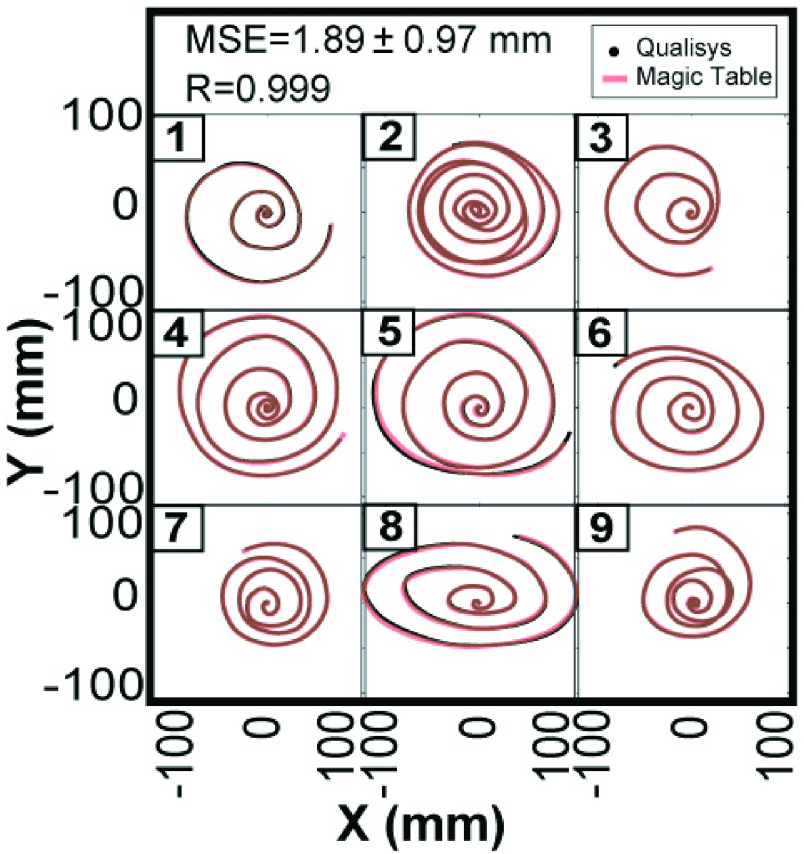
Comparison of recorded data from the MAGIC Table with recordings from 3D motion capture system (Qualisys). The accuracy was quantified by the mean squared error (MSE) and 2D correlation coefficient between the two systems across 9 trials of spiral movements. MSE = 1.89 + 0.97 mm, R = 0.999.

The accuracy of the collected kinematic data by the MAGIC Table was assessed by comparing them with data collected with a camera-based 3D motion capture system (Qualisys, Göteborg, Sweden). For this verification, a subject moved the cup over the whiteboard while performing spiral movements for 9 trials. The curves drawn by the center of the cup were recorded by the two systems; the data from the two systems were filtered by zero-phase digital low-pass filter ($\mathrm {f}_{c}=6$ Hz, 6th order Butterworth), time-synchronized by the alignment of the velocity peaks and resampled for equal resolution. Procrustes analysis reconciled the different coordinate frames from the two systems [32]. Then, the mean squared error (MSE) and 2D correlation coefficient (R) were calculated to serve as a metric for this verification. Fig.6 shows the overlaid traces of 9 trials from the MAGIC Table and the Qualisys recording, illustrating the high degree of overlap. The mean square error was 1.89 ± 0.97 mm calculated over the combined length of 2,790.5 mm in 9 trials; the correlation coefficient was 0.999.

The accuracy of the MAGIC Table recording was further tested by quantifying the effect of possible lens distortion. A conventional camera calibration process in OpenCV using a classical black-white chessboard followed by a distortion correction yielded a minimal or no improvement in MSE and the correlation (1.84 ± 0.89 mm and 0.99, respectively). This suggested that the effect of lens distortion was negligible in the trial data.

### H. Production Costs

The total cost to build the MAGIC Table was less than $200, excluding the price of the computer. The costs included the magnetic whiteboard ($30), a RGB webcam ($55), a folding table with legs ($30), and the parts for the 3D-printed cup ($70).

## III. Feasibility Testing in Pilot Experiments

To demonstrate the MAGIC Table’s effectiveness, this section presents pilot data that was acquired both in a traditional lab settings with two neurotypical children and three children with CP, one of them in an Epilepsy Monitoring Unit (EMU).

### A. Participants

Three children with a clinical diagnosis of hypertonia due to secondary dystonia affecting both hands and two neurotypical children participated in the experiments (Table 1). All participants with dystonia were previously rated on the Barry-Albright Dystonia (BAD) scale [11]. These children were recruited from the movement disorders clinic at the Children’s Hospital of Los Angeles. The two neurotypical children, aged 18 and 19 years, were recruited at the University of Southern California and the laboratory experiments were also conducted at the University of Southern California. The University of Southern California Institutional Review Board approved the study protocol (IRB# UP-12-00457). All participants or their parents gave written informed consent for participation, and all children gave written assent. Authorization for analysis, storage, and publication of protected health information were obtained from parents or participants according to the Health Information Portability and Accountability Act (HIPAA).

**TABLE 1:** Subject Information and Diagnosis. BAD: Upper Extremity Barry-Albright Dystonia Scale. EMU is the Participant Tested in the EMU. N/A: Not Applicable

ID	Diagnosis	Age (yr)	Sex	BAD (L/R)	Tested hand
DY1	Secondary dystonia	18	M	3/3	R
DY2	Secondary dystonia	13	M	3/3	L
EMU	Secondary dystonia	8	M	3/3	L
NT1	Neurotypical	18	M	N/A	R
NT2	Neurotypical	19	F	N/A	R

### B. Experimental Procedures

In the laboratory, four participants (DY1, DY2, NT1, NT2 in Table 1) were seated in front of the MAGIC Table whose height was adjusted for each individual to allow comfortable horizontal arm movements. All participants were reminded to sit with upright posture such that their body did not touch the table. Each participant performed a single experimental session of approximately 1.5 hours. Breaks were given between each block of trials to prevent fatigue.

The clinical experiment was conducted at the Epilepsy Monitoring Unit (EMU) of the Children’s Hospital of Los Angeles. The participant had secondary dystonia and had received surgery for deep brain stimulation (DBS) several days before the assessment (EMU in Table 1). The MAGIC Table whiteboard was placed on an overbed table in the EMU that was also height-adjustable. The tasks were performed with the participant’s upper body elevated to raise the torso into a sitting position. The data collection session lasted about 4 hours, but included several breaks between trials to prevent fatigue. These behavioral data were collected simultaneously with neural recordings from electrodes that were part of the DBS surgery. In addition, muscle activation of the arm muscles was also recorded using electromyography with surface electrodes (DE-2.1, Delsys Inc., USA).

### C. Manipulation of Task Difficulty

**FIGURE 7.**
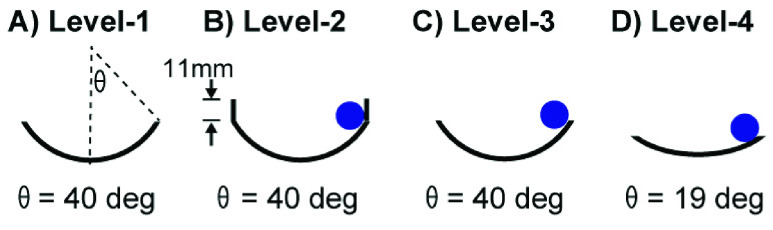
Four levels of difficulty created by the cup design.

For the pilot experiment, four levels of difficulty were created (Fig.7). *Level-1:* This easiest condition involved moving the cup without a ball; the cup radius was 70 mm although the cup radius was irrelevant to the task. *Level-2:* The cup radius was 70 mm and a ball was placed inside the cup. To contain the ball inside the cup, a cylindrical safety rim (height: 11 mm) was placed on top of the perimeter of the dish. The angle at the ball escape was 40 deg. Even though the ball rarely escaped, the movements of the ball made targeted cup movements more difficult than a free movement. *Level-3:* The radius of curvature of the cup was the same as in *Level-2*, but removal of the rim enhanced the difficulty as the ball could easily escape. *Level-4:* The radius of the cup was 140 mm and the angle where the ball escaped was 19 deg.

### D. Point-to-Point Task

The first task involved participants moving from a start circle to a target circle. The targets were located diagonally with respect to the axes of the whiteboard to require flexion and extension movements of the forearm. The longer axis of the rectangular board was aligned with the x-axis and the shorter side with the y-axis. The diameter of the targets were 14 cm at a distance of 52 cm between the two centers of the target. Participants sat in a chair in front of the MAGIC Table and were asked to transport the cup as fast as possible with the rolling ball (where applicable) without dropping. A coin sound served as an auditory feedback when the cup reached the target without losing the ball. Reaching the target successfully was defined when the center of the cup was in the target circle.

After participants gripped the handle they were asked to move the cup between the two target locations as fast as possible. Participants were asked to perform the task with their preferred hand and perform 15 inward (flexion) and 15 outward (extension) trials between two targets. During an inward trial the right-handed subject transported the cup from the top/right target to the bottom/left target with a flexion of the elbow; the outward trials reversed the direction. Each trial was initiated by an auditory go-cue. The experimental task consisted of 4 blocks of 30 trials for each task condition. The order of the levels of difficulty was randomized for each subject. Reach time was defined as the time between the go-cue and when the center of the cup entered the target.

### E. Continuous Figure-8 Task

**FIGURE 8.**
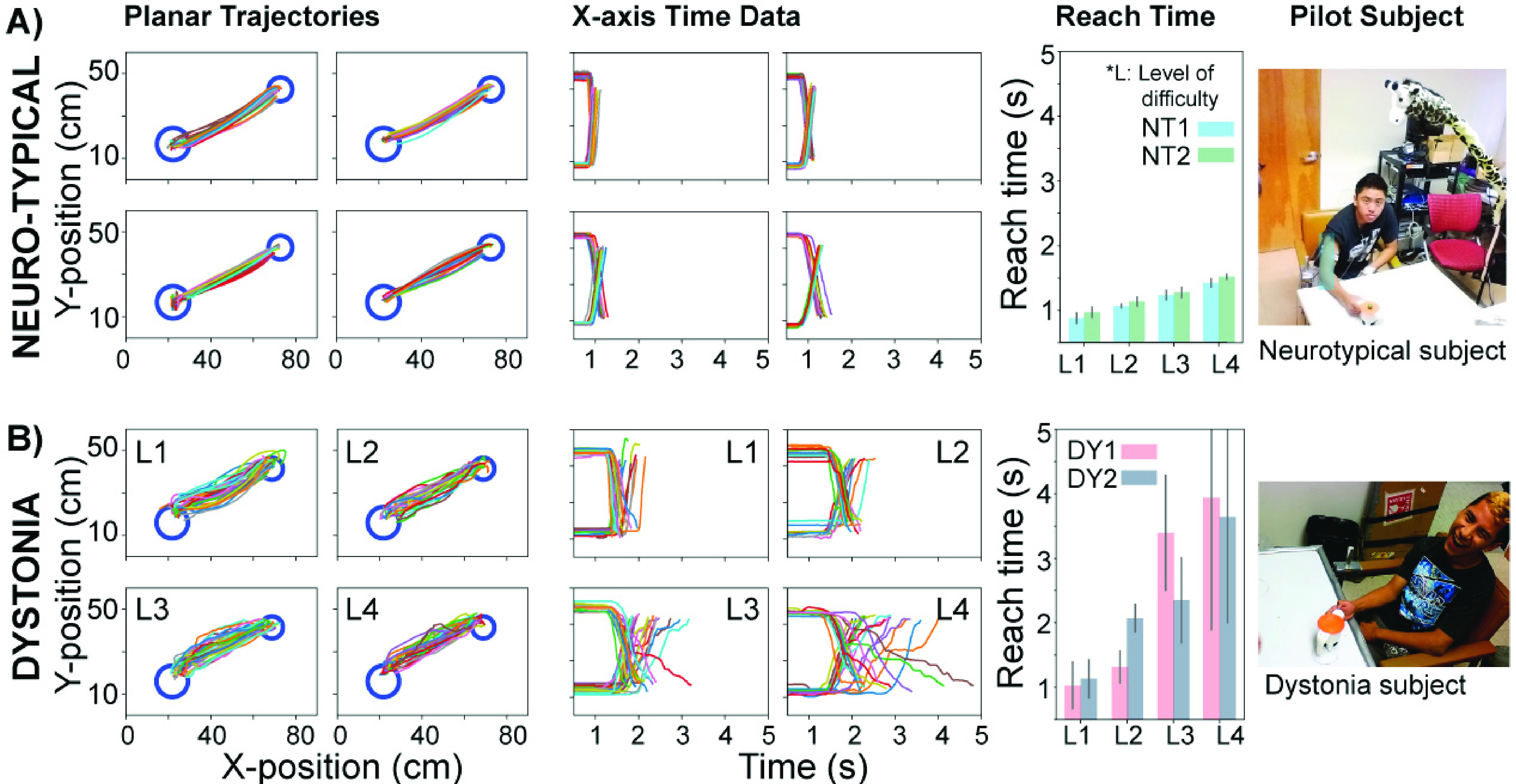
Kinematic trajectories on the horizontal plane, displacement versus time, and reach time as a function of the levels of difficulty (L) of 2 neurotypical and 2 dystonia subjects collected in the laboratory. A: Exemplary trajectory from one neurotypical participant (NT1). For both participants (NT1, NT2), reach time increased as the level of difficulty increased. B: Exemplary trajectory from a participant with dystonia for a discrete task (DY1). For participants with dystonia (DY1, DY2), reach time also increased as the level of difficulty increased.

The participants were asked to move the cup with one hand and track a figure-8 on the whiteboard. The figure-8 was constructed with two circles of 25 cm diameters touching each other. The goal of this task was to accurately track the figure-8. There was no ball as this would have been too difficult. To time the movements, a metronome provided pacing prior to the recording at the participant’s comfortable frequency. The comfortable period for neurotypical participants was 1.66 s; for individuals with dystonia a comfortable period ranged between 3 s and 10 s. For the recording the metronome was turned off. Each neurotypical participant was asked to draw the figure-8 for 3 blocks of 10 trials each. Due to the difficulty of the task, the subjects with dystonia did not complete all the trials and had to terminate their sessions earlier than scheduled.

## IV. Results

### A. Point-to-Point and Figure-8 Movements

Fig.8 shows data from trials from both dystonia participants (DY1, DY2) and neurotypical participants (NT1, NT2) performing the point-to-point task. The exemplary planar position traces (DY1, NT1) show that path variability was considerably higher in the participant with dystonia for all difficulty levels. The exemplary x-position data revealed that reach time was considerably longer for the participant with dystonia than the neurotypical individual. For all available participants (DY1, DY2, NT1, NT2), reach time increased with the level of difficulty L, which was consistent with previous reports [27], [33].

**FIGURE 9.**
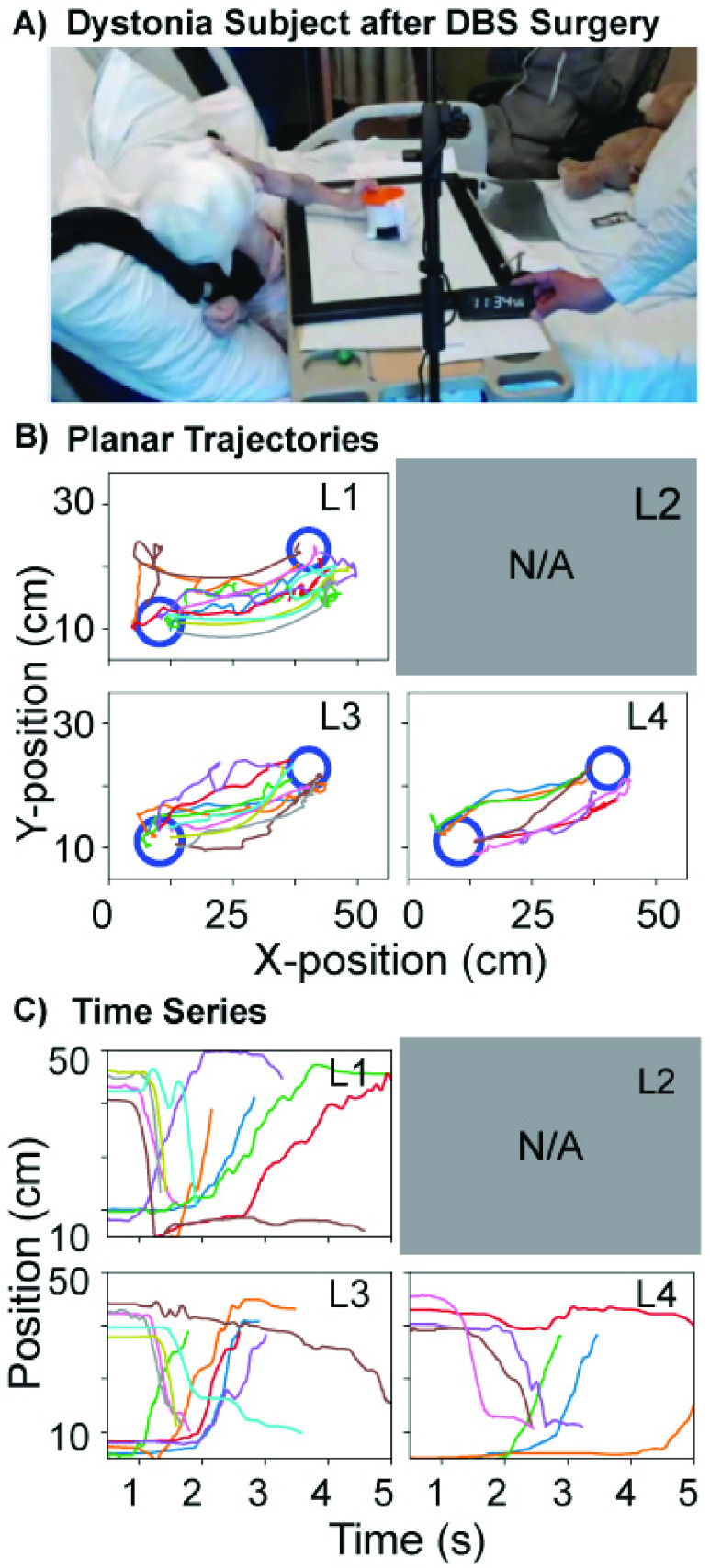
A: The MAGIC Table was placed on an overbed table provided in the hospital on the participant’s bed (EMU). B: Displacement data in the x-y plane of the table. C: Displacement in the x-direction over time. Due to fatigue, the participant only completed three tasks with levels L1, L3, L4.

Fig.9A shows the EMU participant performing the point-to-point movements on an overbed version of MAGIC Table. Fig.9B depicts the planar position traces which had relatively high variability, and the majority of the paths were not straight. The x-displacement over time revealed that reach time also tended to increase with task difficulty seen by the slope that tended to be the steepest at the easiest difficulty level (Fig.9C). Given that the EMU participant was weak following a recent surgery, only a limited number of trials were collected.

**FIGURE 10.**
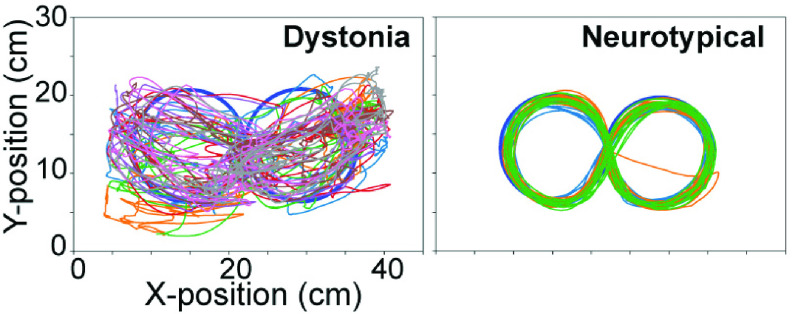
Exemplary trajectories of EMU participant in the figure-8 task over 4 trials (60s each, left). 15s-long trial of a neurotypical subject (NT2, right).

Comparing the figure-8 task performance from the EMU participant with the neurotypical child (NT2) reveals that this task presented visibly more challenge for the EMU participant (Fig.10). The planar position traces show an erratic path of the EMU participant while the neurotypical subjects performed consistent tracking movements.

### B. Synchronized Data Collection With Intracortical Neuron Recordings

**FIGURE 11.**
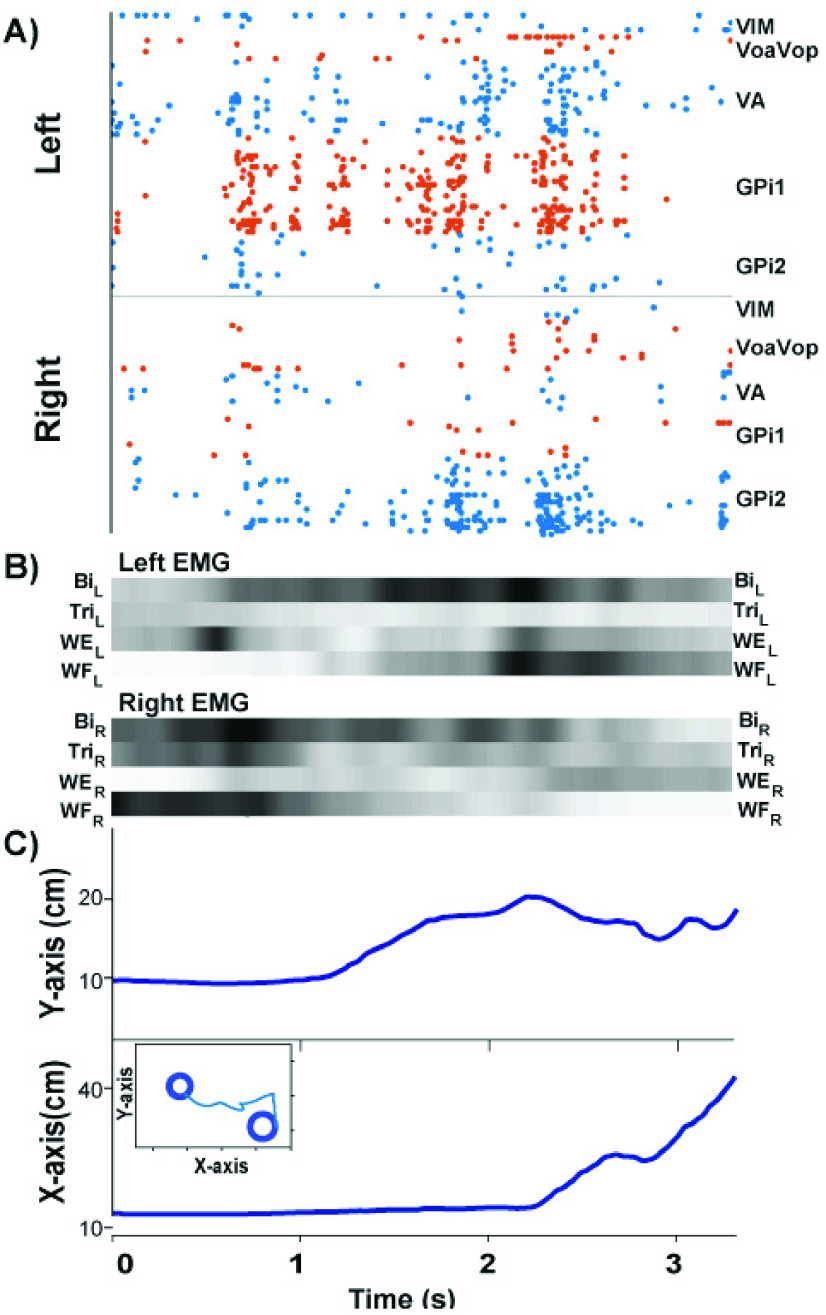
Intracortical neural data synchronized with behavioral data collection in the EMU participant. The neural signals and synchronized kinematics are from a single discrete movement in the outward direction. A: Spike raster recorded from DBS electrodes in the left and right basal ganglia and thalamus. B: EMG signals from the left and right upper extremity displayed in scaled colormap format: the darker the color, the higher the signal intensity. C: Kinematics in the x- and y-direction on the MAGIC Table. The two-dimensional trajectory of the trial is displayed in the inset. Legend: VIM: ventralis intermedius medium. VoaVop: ventralis oralis anterior. ventral oralis posterior. VA: ventralis anterior. GPi: globus palidus internus. Bi: biceps, Tri: triceps, WE: wrist extension. WF: wrist flexor.

To further demonstrate the utility of the MAGIC Table in the clinical setting, we also recorded kinematic data together with intracortical data from the EMU patient, using methods documented in previous case reports [34], [35]. Fig.11 displays a single trial of kinematic data synchronized with the neural recordings from a set of DBS electrodes during the point-to-point movements. Fig.11A shows a spike raster in the thalamus and basal ganglia measured from electrodes with 160 micro and macro contact channels (AD-TECH, Model: MM16C-SP05X-000). The corresponding EMG recordings from 8 muscles of the upper extremity were time-aligned with the kinematics (Fig.11B,C). The subject used his left arm for the task, but the expected higher activity in the left arm is not visible, possibly owing to an overflow or additional effort by the non-task hand. Any interpretation of the neural raster is currently beyond the scope of this paper. We only demonstrate the possibility of time-synchronizing of the kinematic data with simultaneous EMG and neural recordings.

**FIGURE 12.**
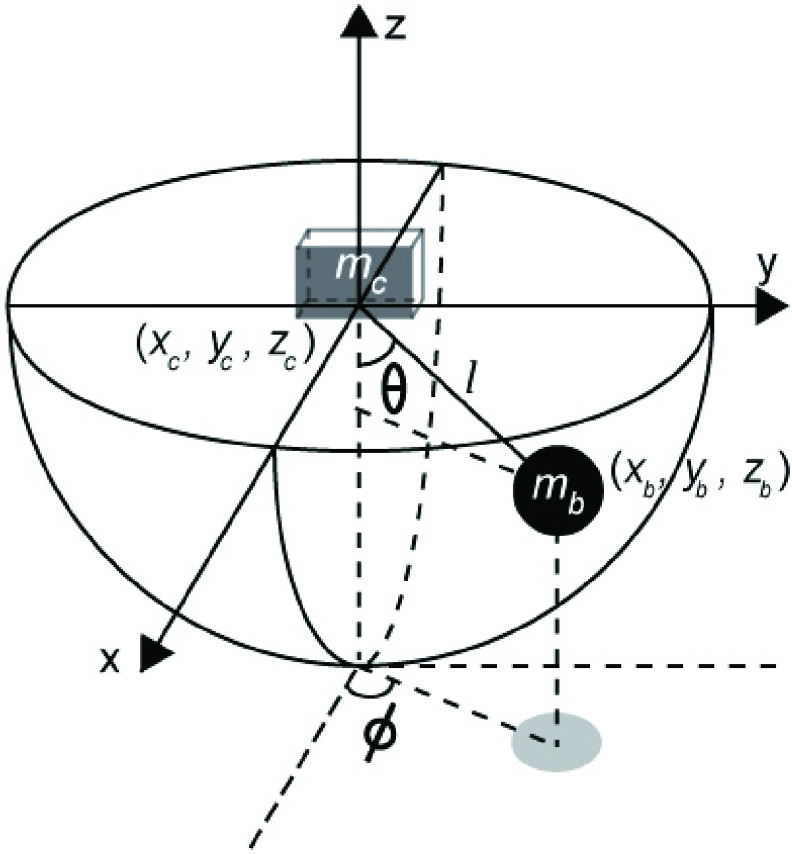
Spherical coordinates of the pendulum. The ball-and-cup system is modeled by a pendulum suspended from a cart. The system is actuated by an external force applied to the cart in the x-y plane. $x_{c}$, $y_{c}$, $z_{c}$: coordinates of the cart, $x_{b}$, $y_{b}$, $z_{b}$: coordinates of the pendulum bob in reference to the cart frame, $\theta $: angle from the negative z axis, l: length of the pendulum, $\phi $: angle from the positive x-axis, $m_{b}$: mass of the bob, $m_{c}$:mass of the cart.

## V. Discussion

There is a paucity of commercially available devices for treatment of upper-limb movement disorders that afford quantitative recording usable for both assessment and rehabilitation of motor function. Existent research prototypes that measure and assist in upper-limb movements are not ideal for home use due to expensive components and sophisticated operation demands. On the other hand, virtual-reality games for CP children, for example based on Wii or Kinect, are entertaining, light-weight, and low-cost, but do neither afford sufficiently precise data recording nor allow for controlled assistance.

This project developed a “game table” for assessment and rehabilitation of upper-extremity movement disorders. With the initial objective to evaluate movements of children with dystonia, we created child-friendly tasks that involved moving a complex object, loosely mimicking the child’s game of transporting an egg in a spoon or a cup filled with a drink [27]. The constraints on the dynamics imposed by the ball and modulated by different curvatures of the cup can add challenge that goes significantly beyond free pointing movements. The design affords titrating and adapting the motor challenges to the patient. The combination of the magnetic table with the custom-designed magnetized object affords a variety of purposeful movement tasks, while also setting a gentle restraint on involuntary movements that may interfere with controlled data acquisition.

Importantly, the MAGIC Table collects precise quantitative kinematic data with low-cost devices that do not require attachment of markers. The data are online analyzed and can provide real-time feedback to the subject. The accurate and continuous sensing affords quantitative research, while its low cost enables use in clinical and home settings. A further unique feature of this device is that it builds on previous basic research on motor control. It therefore can go beyond descriptive error measures and provide metrics that are model-based that are more sensitive than simple outcome measures. The device thereby connects clinical assessment with research.

### A. Kinect-Based Devices and Multi-Object Tracking in Real-Time

Kinect-based systems have been used in many low-cost motion analysis and rehabilitation applications, such as gait assessment for fall detection [36], [37], fall risk reduction in applications for elderly care [38], [39], affordable in-home tele-rehabilitation methods for care and physical rehabilitation [40]–[42], and stroke rehabilitation [43]–[47]. The Kinect’s RGB camera and infrared depth sensors infer body gestures and positions based on the skeletal models obtained via machine learning; its frame rate is up to 30 fps. Despite the excellent accuracy in detecting body gestures and position, it is known that the errors in depth measurement worsen with distance, i.e. grow quadratically with increasing distance from the sensor [48]. Another known underperformance becomes evident in the case of occlusion, or non-participant object interference [49]. Further, if only one camera is used, accurate recordings are largely confined to movements in the frontal plane [50].

The above-mentioned problems are circumvented in the MAGIC Table because its depth tracking (fixed camera distance to the table) only requires a single low-cost RGB camera to track the motion of an object. Distortion can be minimized by using a lens with longer focal length and by further corrections during post-processing. An object with pre-defined shape and color, such as the cup, obviates the need for position inferences by sophisticated machine learning algorithms. Without the need for a depth sensor and sophisticated algorithms, the frame rate can be up to 150 fps as in the current implementation, which is sufficient for acquiring reliable kinematics in typical movement science research. Finally, the design of the cup obviates occlusion since the manipulating hand does not occlude the cup surface from the camera. In addition, the MAGIC Table system enables tracking of multiple objects beyond the end effector, such as the rolling ball in the present case.

The code for data acquisition and processing is easy to use and is provided in the online repository:

(https://github.com/wonjsohn/MAGIC_Table_basic).

It includes functions for camera registration, real-time tracking of circular objects based on color and shape, which are adequate markers for real-time audio feedback based on positions, and frame rate calculation. The code can be expanded to accommodate quantification of various performance metrics depending on the objectives.

### B. Application to Dystonia Patients

The first experiment was conducted with three children with hypertonic dystonia. Hypertonia, together with chorea, tics, and tremor, are examples of positive motor signs that manifest in increased frequency or magnitude of muscle activity, movement, or movement patterns [51]. The magnetic table surface was chosen to gently constrain potential involuntary movements to the planar surface and faciliate quantitative motor assessments. On the other hand, there are negative motor signs, such as weakness, impaired selective motor control, ataxia, and apraxia that manifest insufficient muscle activity or insufficient control of muscle activity. While design features of the MAGIC Table were chosen for individuals with positive signs in mind, inidividuals with negative motor signs may also benefit from practicing with the device.

### C. Assessment and Home-Rehabilitation of Motor Function in Stroke Survivors

We also envisage to use the device for testing motor function in stroke patients with upper-extremity impairments. Such testing is intended to inform treatment plans during the typical stages in post-stroke rehabilitation: 1) therapy during acute hospitalization, 2) therapy for in-patient rehabilitation, 3) home therapy, 4) outpatient therapy or skilled nursing care in a long-term care facility, although not necessarily all of the stages [52].

One core principle of rehabilitation is that recovery and functional gain depends on focused, intense, and repetitive/continuous therapy [53]–[55]. However, such therapeutic interventions are not only labor-intensive, costly, and slow, but are also often challenged by lack of compliance from the patients [56]. For these reasons, an important goal for home-rehabilitation should be to maximize patient compliance and motivation, while maintaining affordability [57]–[59]. The flexibility to create different games by drawing targets or obstacles on the whiteboard with customizable online feedback has the potential to overcome some of these barriers in home-rehabilitation. The MAGIC Table is engaging with an ADL-relevant interface which makes therapy more accessible, affordable and entertaining to result in improved functional outcomes.

Some possible modifications to the device may be needed for other populations such as stroke patients. Depending on the severity of their condition, some patients might require an arm weight support to faciliate horizontal movements on the board. This can be provided in the form or similar to the Freebal system [60]. For other patients holding the cup can be assisted with a wrist support. The device is currently piloted to assess motor performance at post-acute phases of stroke patients at the Stroke Motor Recovery Clinic of Massachusetts General Hospital (MGH).

To summarize, the advantages of the MAGIC Table in application to stroke rehabilitation include the following:

• At low cost, the MAGIC Table can be deployed as in-home rehabilitation device that not only measures the performance for ADL, but also provides a platform for extensive repetitive practice of ADL-related tasks such as drinking, self-feeding, and drawing.
• The MAGIC Table affords tasks that involve range of motion, movement speed, movement smoothness similar to established upper-limb neurorehabilitation devices such as InMotion ARM (Bionik, Toronto, ON Canada), PABLO and DIEGO (Tyromotion GmbH, Graz, Austria). Counter to robotic devices, the MAGIC Table is entirely passive.
• The real task may appeal to older people more than virtual reality devices as they do not require the computer-mediated visuo-motor mapping which can be demanding for people without computer familiarity.
• The automated data acquisition and processing affords assessment of movement quality and feedback of sensitive metrics, such as smoothness or safety margins [23].
• The tasks can be augmented with audio-visual feedback presented on a flat screen. Such multi-sensory feedback or even immersion enhances participation, shown to be effective in neurological rehabilitation [61], [62].
• Frequent motor practice of the stroke-affected limb may counteract the “learned non-use” and prevent the deterioration of motor function.

### D. From Laboratory Research to Clinically Relevant Data

Previous research in our laboratory investigated the strategies that healthy humans employ to interact with the complex dynamics of this object. The focus was on the concepts of stability and predictability and theory-based metrics were derived to evaluate human control strategies [31]. Some of these insights are ripe to be taken into clinical applications [29]. To make the task usable in clinical settings, we designed a real 3D version of the original 2D task. The simultaneous markerless tracking of the positions of the ball and the cup in a real environment will provide rich information about the control strategies of humans in the manipulation of real objects with complex dynamics. Extending the virtual 2D task into 3D also opens the way for more research on modeling and extension of the metrics.

## VI. Conclusion

This study presents an interactive portable motion-analysis device for the assessment and therapy of upper-limb motor functions in a laboratory, clinic and home settings. The clinical utility of the MAGIC Table in motor performance assessment was demonstrated in pilot data from children with dystonia both in the laboratory and at the EMU of the Children’s Hospital of Los Angeles. With the affordability and the ease of replicating the hardware and the open-source software for interested users, there is significant potential for this device to be used in home and clinical assessment and rehabilitation.

## Appendix

### A. The Model

The ball-and-cup system is modeled by a pendulum suspended from a cart. Assuming a reference frame where the cart is at the origin, the coordinates of the two-dimensional pendulum are: the polar angle $\theta $ and the azimuthal angle $\varphi $. The massless rigid rod has an inextensible string of length $l$. The coordinates of the bob of the penduum are:

\begin{align*} x_{b}=&l\sin {\theta \cos \phi } \\
                y_{b}=&l\sin \theta \sin \phi \\ z_{b}=&-l\cos \theta\end{align*}

The two angular coordinates are free to vary independently but $l $ remains invariant. The mass is restricted to move along the surface of the sphere of radius $r = l$. Since the cart is free to move along $x $ and $y$-coordinates, we have a total of four generalized coordinates, $x_{c}$, $y_{c}$, $\theta $, $\phi $ that described the state of the dynamics.

### B. Spherical Pendulum Dynamics

Spherical pendulum dynamics can be obtained based on the energy principle. To this end, one first expresses the difference between the kinetic energy and the potential energy the dynamics known as the Lagrangian $L$. For the pendulum dynamics at hand, the Lagrangian reads:

\begin{align*} L=&\frac {1}{2}\left ({m_{c}+m_{b}
                }\right)\left ({\dot {x_{c}^{2}}+\dot {y_{c}^{2}} }\right) \\&+\,\frac
                {1}{2}m_{b}\Biggl [{ l^{2}\dot {\theta ^{2}}+l^{2}\dot {\phi ^{2}}{\mathrm
                {sin}}^{2}\theta } \\&{+\,2\dot {x_{c}}l\left ({\dot {\theta }\cos \theta \cos
                \phi -\dot {\phi }\sin \theta \sin \phi }\right)} \\&{+\,2\dot {y_{c}}l\left
                ({\dot {\theta }\cos \theta \sin \phi +\dot {\phi }\sin \theta \cos \phi }\right)
                }\Biggr]+m_{b}gl\cos \theta\end{align*}

The above expression can be obtained simply by obtaining the speed of the bob as the time derivatives of the bob positions in three-dimensional coordinates and how much potential energy the bob gains as it swings inside of the sphrere. Next, using ${L}$, one expresses the Euler-Lagrange equations with respect to each generalized coordinate, which consequently provide the equations of motion:

\begin{align*} \frac {d}{dt}\frac {\partial L}{\partial
                \dot {x_{c}}}-\frac {\partial L}{\partial x_{c}}=&\left ({m_{c}+m_{b}
                }\right)\ddot {x_{c}} \\&+\,m_{b}l\Biggl [{ \ddot {\theta }\cos \theta \cos \phi
                } \\& {-\,\left ({\dot {\theta ^{2}}+\dot {\phi ^{2}} }\right)\sin \theta \cos
                \phi } \\&{-\,2\dot {\theta }\dot {\phi }\cos \theta \sin \phi -\ddot {\phi
                }\sin \theta \sin \phi }\Biggr]=F_{x} \\ \frac {d}{dt}\frac {\partial L}{\partial
                \dot {y_{c}}}-\frac {\partial L}{\partial y_{c}}=&\left ({m_{c}+m_{b}
                }\right)\ddot {y_{c}} \\&+\,m_{b}l\Biggl [{ \ddot {\theta } cos \theta sin \phi
                } \\&{-\,\left ({\dot {\theta ^{2}}+\dot {\phi ^{2}} }\right) sin \theta sin
                \phi } \\&{-\,2\dot {\theta }\dot {\phi } cos \theta cos \phi -\ddot {\phi } sin
                \theta cos \phi }\Biggr]=F_{y} \\ \frac {d}{dt}\frac {\partial L}{\partial \dot
                {\theta }}-\frac {\partial L}{\partial \theta }=&m_{b}l\Biggl [{ l\ddot {\theta
                }+\ddot {x_{c}} cos \theta cos \phi +\ddot {y_{c}} cos \theta sin \phi } \\&
                {-\,l\dot {\phi ^{2}} sin \theta cos \theta +g sin \theta }\Biggr]= 0 \\ \frac
                {d}{dt}\frac {\partial L}{\partial \dot {\phi }}-\frac {\partial L}{\partial \phi
                }=&m_{b}l\Biggl [{ l\ddot {\phi }{sin}^{2}\theta -2l\dot {\phi }\dot {\theta }
                sin \theta cos \theta } \\& {-\,\ddot {x_{c}} sin \theta sin \phi +\ddot {y_{c}}
                sin \theta cos \phi }\Biggr]=0\end{align*}

The input forces $F_{x}$ and $F_{y}$ can be measured or simulated to generate cup and ball trajectories. Further, based on kinematic measurements, more metrics can be derived. For example, the distance of the ball to the rim, or the safety margin, can be calculated based on the ball kinematics and the knowledge of the radius of curvature, i.e. pendulum length, of the cup. These equations can also be used to implement this object into a virtual environment.
